# Nutritional and speech therapy guidance for the introduction of foods in infants: dietary profile and risk of pediatric feeding disorder

**DOI:** 10.1590/2317-1782/e20240377en

**Published:** 2026-03-27

**Authors:** Larissa Barz de Vargas, Giane Eichner, Bianca Nunes Pimentel, Giovana Cristina Ceni, Geovana de Paula Bolzan

**Affiliations:** 1 Programa de Pós-graduação em Distúrbios da Comunicação Humana, Universidade Federal de Santa Maria – UFSM - Santa Maria (RS), Brasil.; 2 Curso de Fonoaudiologia, Universidade Federal de Santa Maria – UFSM - Santa Maria (RS), Brasil.; 3 Departamento de Alimentos e Nutrição, Universidade Federal de Santa Maria – UFSM - Santa Maria (RS), Brasil.; 4 Departamento de Fonoaudiologia, Universidade Federal de Santa Maria – UFSM - Santa Maria (RS), Brasil.

**Keywords:** Feeding Behavior, Infant, Infant Nutrition, Diet, Nutrition Assessment

## Abstract

**Purpose:**

To assess the dietary profile, nutritional status and risk of pediatric eating disorders in healthy infants undergoing food introduction, and to compare these variables between groups that received or did not receive specialized nutritional and speech therapy guidance.

**Methods:**

Descriptive, observational, cross-sectional study with a quantitative approach. The Guided Group was composed of infants whose parents reported having received guidance on food introduction in a multidisciplinary outpatient clinic. The Non-Guided Group was composed of infants whose parents did not receive guidance on food introduction or received it in childcare by other professionals. Medical records were analyzed and interviews and anthropometric measurements were taken to outline the clinical and nutritional profile. Diet quality was assessed using the Food Consumption Markers Form. The classification of nutritional status followed the World Health Organization curves. The risk of pediatric eating disorders was assessed using the Brazilian Infant Feeding Scale.

**Results:**

Sixty infants participated. The Non-Guided Group started complementary feeding early, before six months. The quality of the Guided Group's diet was better, with greater dietary diversity, higher consumption of foods rich in vitamin A and lower intake of ultra-processed foods. There was a predominance of eutrophy in both groups. The Guided Group had a lower risk of pediatric eating disorders.

**Conclusion:**

Nutritional and speech therapy guidance for the initiation of complementary feeding was associated with the quality of the infants' diet, but was not associated with nutritional status. The risk of pediatric eating disorders was more frequent in the Non-Guided Group.

## INTRODUCTION

Nutrition plays an extremely important role in all phases of life, especially in the first two years. During this period, it is essential to encourage and adopt healthy eating habits, which are crucial for providing full growth and development^(1)^. The dietary pattern established in this age group tends to remain and consolidate into adulthood. Adequate food consumption, in addition to ensuring nutritional needs, will reduce the risk of morbidity, mainly malnutrition, overweight, micronutrient deficiency and associated diseases^(2)^.

The World Health Organization (WHO) recommends exclusive breastfeeding until six months of age and continued breastfeeding until two years or more, because breast milk is unparalleled and fully adapted to the needs of the child in the first months of life^(2-4)^.

However, from six months onwards, feeding should be supplemented. The nutritional quality of complementary foods is fundamental in preventing morbidity and mortality in childhood. The recommendation to start introducing food at six months takes into account the physiological and neuromuscular maturity required for the infant to receive solid foods^(3,4,5,6)^. At this age, the child begins to establish food preferences. In addition, it is possible to observe the disappearance of the tongue protrusion reflex, better gastrointestinal tolerance and better nutrient absorption capacity^(2)^. Early complementary feeding can lead to renal overload, food allergies, nutritional deficiencies, changes in intestinal flora, intestinal and respiratory infection and obesity. Late introduction of food can also have consequences such as growth retardation and immunological deficiencies^(2,7)^.

The Food Guide for Brazilian children under two years of age, published by the Ministry of Health (MS)^(3)^, reinforces and disseminates the importance of healthy eating, emphasizing unprocessed or minimally processed foods as the basis of the child's and the whole family's diet, in addition to guiding the appropriate consistency according to age group. Despite this, authors have highlighted the high consumption of ultra-processed foods among children under one year of age, in addition to the offering of foods in an inappropriate consistency according to the age group, which may cause negative repercussions in the following stages of development^(8)^.

Nutritional status indicates the result of the balance between nutrient consumption and the body's energy expenditure. Anthropometric assessment is considered a good indicator of health, due to its ease of application and standardization. In addition, the low cost and breadth of aspects analyzed allows for the mapping of individual or collective nutritional profiles^(9)^. Assessment of nutritional status in the first year of life is fundamental, since accelerated growth occurs during this period^(10)^. Early identification of infants at risk, with deficits or excesses in their growth, becomes essential, as nutritional factors directly influence metabolism and can lead to undesirable long-term consequences^(3,11)^.

Pediatric feeding disorder (PFD) is considered a complex and multifactorial condition. It has been defined as impaired oral intake, which is not appropriate for the age, associated with at least one of the following dysfunctions: medical, nutritional, feeding skills and/or psychosocial^(12)^. This disorder can occur at any stage of childhood, being more prevalent around six months to four years of age^(12-13)^. Studies indicate a prevalence of PFD of 20% to 35% among children with normal intellectual development^(14-15)^.

Due to its complexity, an interdisciplinary team is essential for the diagnosis, treatment and monitoring of PFD^(14)^. It is worth noting that the act of eating transcends anatomical and physiological issues, as it is embedded in a social and cultural context, thus being associated with social, emotional and cognitive growth and development. Early identification of this disorder by health professionals can minimize and solve eating problems through early and specialized treatment^(15)^.

The period of introducing complementary feeding has repercussions in all phases of life; however, few studies have been conducted regarding the feeding and nutrition of infants during the introduction of food. Thus, the objective of this study was to evaluate the dietary profile, nutritional status, and risk of foodborne illness in healthy infants during the introduction of food, as well as to compare these variables between groups that received or did not receive specialized nutritional and speech therapy guidance.

## METHOD

This is a descriptive, observational, cross-sectional study with a quantitative approach. The research project was previously approved by the ethics committee of the institution of origin under opinion number 5,861,862.

The study was conducted with full-term infants, followed up in a multidisciplinary pediatric outpatient clinic at the University Hospital of Santa Maria, a tertiary-level hospital that serves as a reference for the central region of Rio Grande do Sul. Infants aged seven to twelve months who had already started complementary feeding for at least one month and who were exclusively fed orally were included in the sample. Infants diagnosed with neurological or cardiological diseases, craniofacial or aerodigestive tract malformations, or any other clinical condition that directly interfered with the safety of swallowing and the effectiveness of oral feeding were excluded. All participants had the Informed Consent Form signed by their guardians. Data collection was carried out from October 2022 to September 2023.

The sample size calculation was performed based on a pilot study with ten subjects seen at the infant feeding clinic. The parameter used for this calculation was the T-score of the Brazilian Infant Feeding Scale. A significance level (α) of 95% and a sampling error of 5% were considered, resulting in a minimum sample size of 60 infants, allowing comparisons between the subgroups formed according to exposure to specialized guidance. The subsequent categorization of participants into two subgroups, guided and unguided, was carried out based on the information obtained during data collection, and served for analytical and comparative purposes. The Guided Group consisted of infants whose parents received specialized nutritional and speech therapy guidance for food introduction. The Unguided Group consisted of infants whose parents started food introduction on their own or as guided in childcare, by a doctor or nurse. Comparisons between groups sought to identify associations between specialized guidance and the variables studied, such as diet quality, nutritional status, and risk for PFD, in a consistent manner that does not allow for inferences of causality. Exposures and outcomes were collected simultaneously, and comparisons were made based on the conditions observed at the time of collection.

The sample was recruited from the hospital's multidisciplinary pediatric outpatient clinic. Caregivers were invited to participate in the study by telephone, and in-person appointments were scheduled at the hospital. Families who had received specialized care for introducing solid foods at the outpatient clinic were invited to participate in the Guided Group. Families who had been seen at the outpatient clinic for other reasons, such as lingual frenulum assessment; hearing assessment; clinical management of breastfeeding at birth; and participation in the musical intervention group for infants, and who had not received speech therapy and/or nutritional care for complementary feeding, were invited to participate in the Non-Guided Group.

The care for guidance on introducing solid foods, in which the infants in the Guided Group participated, is routinely provided at the multidisciplinary outpatient clinic. This specialized care is offered to all families who come for care soon after the birth of their babies, and they have the option of scheduling the appointment. The guidance is provided to the parents of infants, jointly by a nutritionist and speech therapist and/or a Speech Therapy student under academic supervision. The nutritional and speech therapy guidance provided in these sessions is based on the book Cognitive Relationships with Food in Childhood^(16)^ and the Food Guide for Brazilian children under two years of age^(3)^. The following topics are covered: continued breastfeeding; food groups, meal fractionation; amount of food to be offered; signs of hunger and satiety; myths and beliefs about complementary feeding; consequences of early or late introduction of food; methods of offering food (traditional, BLW (Baby-Led Weaning) and mixed); appropriate food consistency; appropriate utensils; child positioning; as well as family interaction at the time of food offering. The guidance is explained orally and delivered in writing, in material containing text and images. In addition, participants receive contact information for a permanent channel to ask questions of the professionals, if needed, via social media.

Data collection involved investigating clinical and sociodemographic data, applying screening protocols for food consumption profile, nutritional assessment, and risk assessment for PFD. First, information was collected from medical records regarding the following variables: age (in months), sex (female or male), race (white and non-white), mother's age (in years), birth weight (kg), and height (cm). Subsequently, an interview was conducted to collect data on family income, support network, breastfeeding, age at which food introduction began, whether the caregiver received guidance from any professional regarding food introduction, type of food initially offered, and whether the child experienced any difficulties with food introduction.

The quality of infants' diet was assessed using the MS Food Consumption Markers Form for children under two years of age. This instrument aims to understand the pattern of food consumption through questions applied to parents according to the infant's age group. The questions allow us to characterize the introduction of complementary feeding, identify the type of current diet, and verify the adoption of dietary behaviors that pose a risk for the development of anemia and childhood obesity ^(17)^.

The food consumption variables were organized based on the main food groups and risk behaviors identified in the form. The food groups include fruits, vegetables, meats, grains, and dairy products, categorized according to the presence or absence of consumption on the day before the survey. In addition, the frequency of daily intake and the form of offering the food is considered (example: in pieces, mashed, passed through a sieve, blended, or just the broth). Regarding risk behaviors, variables such as consumption of sugars, sweetened juices, and ultra-processed foods were identified, in addition to the intake of foods rich in iron, categorized as presence or absence. These variables allow for a comprehensive characterization of the quality of the diet and the foods.

The nutritional status assessment was performed by an experienced nutritionist. Weight was measured using a digital pediatric scale (Balmak®) with 0.010 kg graduation and a capacity of 15 kg, and length was measured using an infant stadiometer (Avanutri®) with 1 mm graduation. The 2006 World Health Organization growth charts^(18)^ were used to classify the nutritional status of infants, using the Who Anthro software, according to the Z-score.

To assess the risk of PFD, the Brazilian Infant Feeding Scale (EBAI) was applied. This screening instrument is intended for children aged 6 months to 5 years and 11 months and has recently been validated for the Brazilian population. The EBAI consists of fourteen questions related to the child's feeding, directed to caregivers. The questions cover the feeding domains: oral motor, oral sensory, appetite, and parental concerns about feeding. The presence or absence of PFD risk is calculated using a score at the end of the questionnaire. When the total score is up to 60, it is considered an absence of PFD risk; scores from 61 to 65 indicate mild risk; scores from 66 to 70 indicate moderate risk; and scores above 70 indicate severe risk^(14)^.

All screening instruments were applied by a nutritionist and a speech therapist, previously trained. The data were entered into a spreadsheet using Excel® version 2010 for Windows and subsequently exported to Statistica version 9.1. Analysis of the studied variables was performed, presenting means and standard deviations or proportions, according to the nature of the variable. Association analyses were performed using Pearson's chi-square or Fisher's exact tests. The Student's t-test was used to verify differences in means. The significance level for all analyses was 5% (p<0.05).

## RESULTS

Sixty full-term infants participated in the study. The mean age of the sample was 8.95 ± 1.75 months. The majority were male (63.3%), white (80%), and lived in families with a support network (85%). The mean gestational age at birth was 38.1 ± 1.21 weeks, and the mean maternal age was 30.3 ± 6.7 years. The mean per capita income of the sample was R$ 627.73 ± 252.60.

When stratified by reported receipt of specialized nutritional and speech therapy guidance for the introduction of food, it was observed that the Guided Group had a mean age of 9.4 ± 2.04 months, while the Non-Guided Group had a mean age of 8.5 ± 1.30 months (p = 0.018). Although a statistically significant difference in age was identified between the subgroups, multivariate analysis was not performed in this study due to the sample size. However, the proximity between the means (9.4 vs. 8.5 months) suggests that this age difference does not compromise the interpretation of the observed results.

Table 1 details the demographic, socioeconomic, and family support characteristics of the total sample and according to reports of receiving specialized guidance.

**Table 1:** Demographic, socioeconomic and family support characteristics of the total sample and according to reports of receiving specialized guidance (N = 60)

**Variables**	**Groups**				
	**Oriented (N=30)**		**Not oriented (N=30)**		
	**N (%)**	**Mean ± SD**	**N (%)**	**Mean ± SD**	**p**
**Gestational age at birth (weeks)**		37.88 ± 1.14		38.33 ± 1.26	0.605*
**Current age (months)**		9.40 ± 2.04		8.50 ± 1.30	0.018*
**Maternal age**		30.13 ± 6.88		30.46 ± 6.68	0.875*
**Per capita income (Brazilian real)**		638.00 ± 241.41		617.46 ± 264.94	0.619*
**Sex**					
Female	9 (30.00)		13 (43.33)		0.283**
Male	21 (70.00)		17 (56.67)		
**Color**					
White	25 (83.33)		23 (76.67)		0.518**
Not white	5 (16.67)		7 (23.33)		
**Support network**					
With	28 (93.33)		23 (76.67)		0.072**
Without	2 (6.67)		7 (23.33)		

* Student's t-test;

** Pearson's Chi-square test

Caption: SD = Standard Deviation; N = number of participants; % = percentage of participants

Regarding complementary feeding, the average age of initiation was 6.16±0.46 months in the Guided Group and 5.46±1.10 months in the Non-Guided Group. Table 2 shows the dietary characteristics of infants during the food introduction phase. Mixed feeding predominated in the Guided Group and artificial feeding in the Non-Guided Group when complementary feeding began. Fruits and savory foods were the foods chosen to be offered initially, and no difficulties were found in introducing complementary feeding in either group. It was found that 63.33% of the families of infants in the Non-Guided Group did not receive specific guidance regarding the introduction of complementary feeding.

**Table 2:** Dietary characteristics of infants during the food introduction phase according to reports received from guidance (N = 60)

**Variables**	**Groups**		
	**Oriented (N=30)**	**Non Oriented (N=30)**	
	**N (%)**	**N (%)**	**p**
**Type of food initially offered**			
Fruit	10 (33.33)	7 (23.33)	
Salty food	11 (36.67)	11 (36.67)	0.843**
Fruits and salty food	11 (36.67)	12 (40.00)	
**Professional who guided the introduction of solids**			
Nutritionist and speech therapist	30 (100.00)	0 (0.00)	0.001**
Other professional in childcare	0 (0.00)	11 (36.66)	
No guidance received	0 (0.00)	19 (63.33)	
**Difficulties with introduction of solids**			
Yes	12 (40.00)	13 (43.33)	0.793**
No	18 (60.00)	17 (56.67)	
**Type of breastfeeding until IA**			
AA	9 (30.00)	19 (63.33)	
ABM	20 (66.67)	9 (30.00)	0.017**
EBF	1 (3.33)	2 (6.67)	

** Pearson's Chi-square test

Caption: N = number of participants; % = percentage of participants; IA = introduction of food; AA = artificial feeding; ABM = mixed breastfeeding; EBF = exclusive breastfeeding

The quality of complementary feeding in the Guided Group was significantly better compared to that of the Non-Guided Group. The Guided Group had greater dietary diversity and higher consumption of foods rich in vitamin A (vegetables, vegetables or orange-colored fruits), while the Non-Guided Group had high consumption of ultra-processed foods (Table 3).

**Table 3:** Quality of nutrition during the introduction of solid foods for infants, assessed by food consumption markers, according to reports of guidance received (N = 60)

**Variables**	**Groups**		
	**Oriented** **(N=30)**	**Non Oriented (N=30)**	
	**N (%)**	**N (%)**	**p**
**General Nutrition**			
Adequate	24 (80.00)	21 (70.00)	0.001**
Inadequate	6 (20.00)	9 (30.00)	
**Continued breastfeeding**			
Yes	18 (60.00)	13 (43.33)	0.196**
No	12 (40.00)	17 (56.67)	
**Introduction of solid foods**			
Yes	19 (63.33)	16 (53.33)	0.432**
No	11 (36.67)	14 (46.67)	
**Minimum dietary diversity**			
Yes	22 (73.33)	13 (43.33)	0.018**
No	8 (26.67)	17 (56.67)	
**Minimum feeding frequency**			
Yes	22 (73.33)	21 (70.00)	0.774**
No	8 (26.67)	9 (30.00)	
**Consumption of iron-rich foods**			
Yes	27 (90.00)	23 (76.67)	0.149***
No	3 (10.00)	7 (23.33)	
**Consumption of vitamin A-rich foods**			
Yes	28 (93.33)	20 (66.67)	0.010***
No	2 (6.67)	10 (33.33)	
**Consumption of ultra-processed foods**			
Yes	6 (20.00)	23 (76.67)	0.001**
No	24 (80.00)	7 (23.33)	
**Hamburgers/cured meats**			
Yes	0 (0.00)	22 (73.33)	0.001***
No	30 (100.00)	8 (26.67)	
**Sweetened beverages**			
Yes	2 (6.67)	10 (33.33)	0.010***
No	28 (93.33)	20 (66.67)	
**Instant noodles, packaged snacks and savory biscuits**			
Yes	1 (3.33)	11 (36.67)	0.001***
No	29 (96.67)	19 (63.33)	
**Filled biscuits, sweets and candies**			
Yes	4 (13.33)	18 (60.00)	0.001***
No	26 (86.67)	12 (40.00)	

** Pearson's Chi-square test;

*** Fisher's Exact Test

Caption: N = number of participants; % = percentage of participants

**Figure 1:**
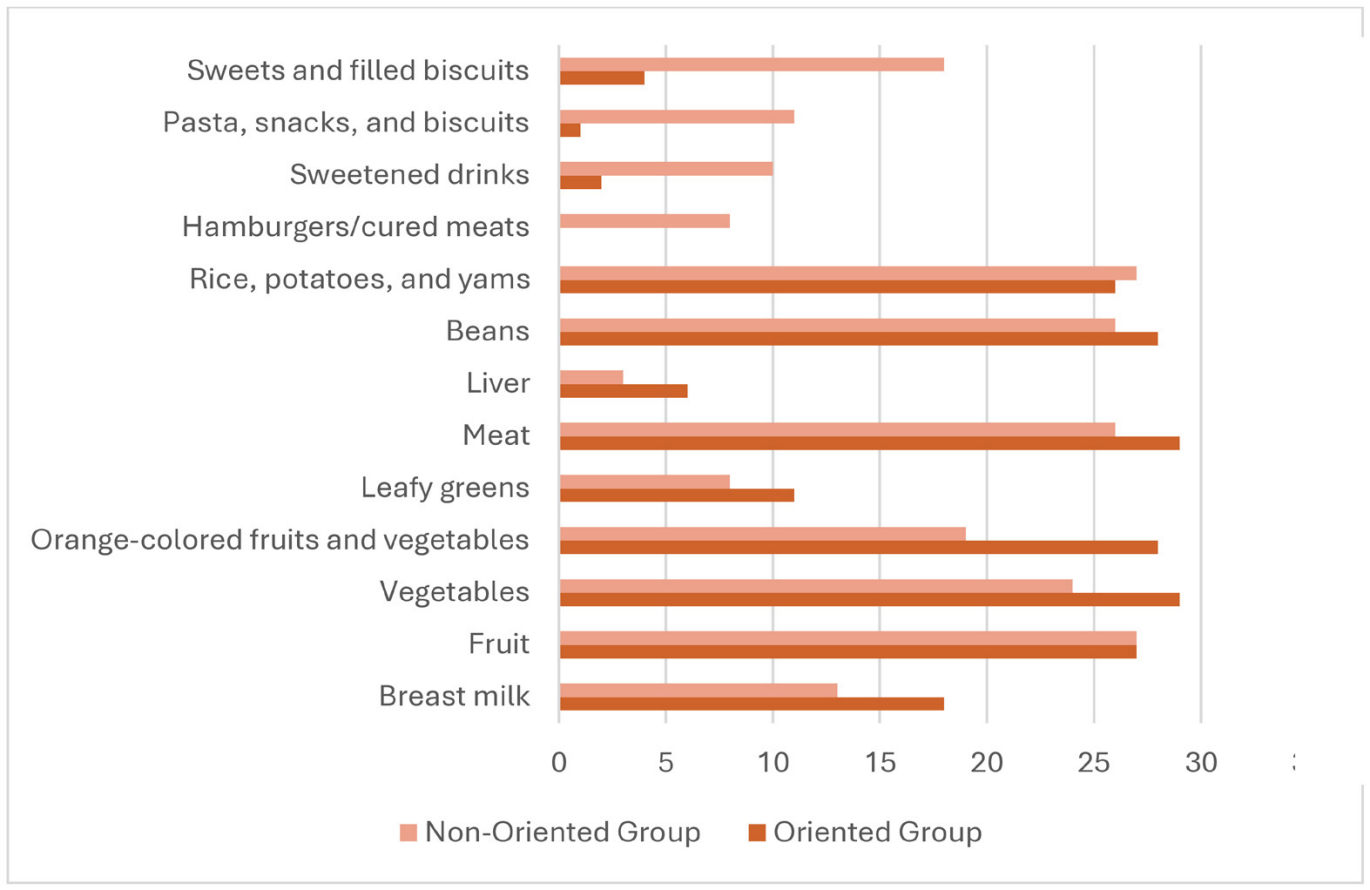
Dietary pattern of the sample, according to the groups

Figure 1 shows a significant difference in food consumption, where the Guided Group had a higher consumption of unprocessed or minimally processed foods (rice, potatoes, yams, beans, liver, meats, vegetables and legumes) and the Non-Guided Group had a higher intake of ultra-processed foods (sweets and filled biscuits; pasta, snacks and biscuits; sweetened beverages and hamburgers/processed meats).

Regarding nutritional status (Table 4), presented as a Z-score, the prevalence of eutrophy at birth and at the time of data collection was observed, according to the weight-for-height indicator, in both groups.

**Table 4:** Anthropometric characteristics and nutritional status of infants at birth and during the introduction of solid foods, according to reports of guidance received (N = 60)

**Variables**	**Groups**				
	**Oriented (N=30)**		**Non Oriented (N=30)**		
	**N (%)**	**Mean ± SD**	**N (%)**	**Mean ± SD**	**p**
Birth weight (kg)		2.96 ± 0.54		3.06 ± 0.44	0.253*
Birth height (cm)		46.92 ± 2.65		47.97 ± 2.32	0.489*
Current weight (kg)		8.85 ± 1.32		8.96 ± 1.29	0.909*
Current height (cm)		71.50 ± 4.53		73.48 ± 5.66	0.242*
**Weight for birth height**					
Severe thinness	8 (26.67)		4 (13.33)		0.353**
Thinness	1 (3.33)		0 (0.00)		
Normal weight	13 (43.33)		21 (70.00)		
Risk of overweight	5 (16.67)		5 (16.67)		
Overweight	2 (6.67)		0 (0.00)		
Obesity	1 (3.33)		0 (0.00)		
**Weight for current height**					
Severe thinness	0 (0.00)		1 (3.33)		0.891**
Thinness	0 (0.00)		3 (10.00)		
Normal weight	26 (86.21)		23 (76.67)		
Risk of overweight	1 (3.45)		3 (10.00)		
Overweight	3 (10.34)		0 (0.00)		
Obesity	0 (0.00)		0 (0.00)		
**Weight at birth age**					
Very low	5 (16.66)		0 (0.00)		
	1 (3.33)		5 (16.66)		0.600**
	24 (80.00)		25 (83.33)		
	0 (0.00)		0 (0.00)		
Low					
	0 (0.00)		0 (0.00)		
	1 (3.33)		1 (3.33)		0.561**
	25 (83.33)		28 (93.33)		
	3 (10.00)		1 (3.33)		
Suitable					
	0 (0.00)		0 (0.00)		
	3 (10.00)		0 (0.00)		0.705**
	27 (90.00)		30 (0.00)		

* Student's t-test;

** Pearson's Chi-square test

Caption: SD = Standard Deviation; N = number of participants; % = percentage of participants

A higher frequency of risk of PFD was identified in the Non-Guided Group, classified as mild to moderate risk (Table 5).

**Table 5:** Association of risk of pediatric eating disorder in infants with groups and frequency of risk classification in the study and control groups (N = 60)

**Variables**	**Groups**		
	**Oriented (N=30)**	**Non Oriented (N=30)**	
	**N (%)**	**N (%)**	**p**
**Risk of PFD**			
Yes	1 (3.33)	10 (33.33)	0.001**
No	29 (96.67)	20 (66.67)	
**PFD risk classification**			
No risk	29 (96.67)	20 (66.67)	-
Mild	1 (3.33)	7 (23.33)	
Moderate	0 (0.00)	3 (10.00)	
Severe	0 (0.00)	0 (0.00)	

** Pearson's Chi-square test

Caption: N = number of participants; % = percentage of participants; PFD = Pediatric feeding disorder

## DISCUSSION

According to the Ministry of Health, the introduction of complementary foods should begin at six months of age, slowly and gradually. During this period, the infant's energy and nutrient needs exceed what can be provided by exclusive breastfeeding, and the infant begins to show signs of readiness, which allow the introduction of food. Complementary feeding has an extremely relevant relationship in the physical, neurological and motor development of the child and has repercussions on the formation of healthy eating habits, which persist into adulthood^(3)^.

In this study, a higher frequency of artificial feeding and early initiation of complementary feeding was observed in the Non-Guided Group. This finding corroborates what was evidenced in a retrospective study, whose authors, when characterizing the complementary feeding of more than 700 infants, found that the average age for the introduction of food was 5.5 months^(19)^. Carneiro et at.^(20)^ also observed, in their study, that 66.66% of infants started complementary feeding early, between four and five months of age, and the reasons given by parents for this start were: the use of infant formula, return to work, low weight gain and maternal choice. Authors^(6)^ state that parents of infants choose to offer other foods, feeling safer because they have greater control over what the child is ingesting. The explanation for this would be the lack of guidance or understanding about the physiological and hormonal processes of breast milk production and also because they cannot control the amount of breast milk ingested^(6)^.

However, the early introduction of complementary foods can cause a number of problems. Initially, this practice leads to a decrease in breastfeeding, which can lead to the interruption of balanced oral-motor development that occurs during breastfeeding, with possible consequences for the functions of breathing, chewing, swallowing and speech^(2)^. From a nutritional point of view, the early introduction of complementary feeding can interfere with the absorption of important nutrients obtained from breast milk, and may also lead to an increased risk of contamination, allergic reactions and atopic diseases^(11)^. Furthermore, early nutritional experience can have a lasting effect, persistent throughout life, predisposing the subject to certain diseases^(11)^.

In this study, nutritional and speech therapy guidance was associated with better indicators in the introduction of complementary feeding. Infants whose parents were guided in specific care for this purpose, the Guided Group, started food introduction at the recommended age, showing better diet quality and a lower risk of PFD. The nutritionist and speech therapist, as well as other health professionals, play an important role in advising families, since the child's eating behavior is a consequence of the parents' attitudes^(21)^. Timely health education, reinforcing the superiority of breast milk and highlighting the importance of proper food introduction, using playful and dynamic strategies, provides families with greater autonomy and the formation of healthy eating habits in children^(22)^.

Regarding the quality of infants' diets, according to the food consumption markers proposed by the Ministry of Health^(17)^, the Guided Group showed a higher frequency of food diversity (more than four food groups) and higher consumption of foods rich in vitamin A, characterizing a more adequate dietary pattern. Similar results were observed by other authors who developed a randomized controlled trial with infants aged six to 12 months and their mothers^(23)^. They observed that the group that received nutritional education focusing on complementary feeding showed greater food diversity^(23)^. These findings support the importance of nutritional guidance for infants, since they may present a number of nutritional deficiencies, mainly iron deficiency anemia. And, in addition, they reinforce the need to provide micronutrients from complementary feeding^(2)^.

It is suggested that the positive relationship of the guidance provided to the Guided Group may have occurred due to the welcoming and guidance given to the infants' parents on how to proceed during the start of complementary feeding. It is also worth highlighting that the clinic where the guidance is provided has a digital channel where families can resolve their doubts, which may have contributed to the safety of family members at the time of food introduction. According to Vieira et al*.* ^(24)^, dialogue provides the building of bonds with students, facilitating interaction in the teaching-learning process, contributing to the dissemination of knowledge about healthy eating and resulting in good adherence to the guidelines.

In contrast, in the Non-Guided Group, the early introduction of ultra-processed foods was observed, including: hamburgers/sausages, sweetened beverages, instant noodles, packaged snacks, savory biscuits, filled biscuits, sweets and candies, showing a significant difference between the groups, which may be associated with the contemporary dietary pattern. It is worth highlighting that the Ministry of Health’s Food Guide for children under two years old^(3)^ recommends the minimum addition of salt in the preparations offered and the introduction of foods rich in sugar or preparations with added sugar only from two years of age^(3)^. The intake of foods with high rates of calories, sodium, sugars and fats generates short- and long-term consequences, being associated with excess weight, dental caries, micronutrient deficiency, dyslipidemia, high blood pressure and diabetes^(1)^.

In the same direction, other studies have shown the prevalence of early introduction of foods with high caloric density and low nutritional quality^(25)^, and pointed out that more than half of children before one year of age ingested sweets, chocolate drinks and instant noodles^(11)^, in addition to the high consumption of fried foods, soft drinks and salt^(1)^. Data from the Food and Nutrition Surveillance System (SISVAN) in 2022 also demonstrated the early consumption of ultra-processed foods by children between six and 23 months of age, with 44% consuming ultra-processed foods, 12% processed foods, 22% instant noodles, savory snacks or biscuits, 25% filled biscuits, sweets or candies and 28% sweetened beverages^(26)^.

Regarding nutritional status, it was observed that both groups, at birth and at data collection, presented a prevalence of eutrophy, with no anthropometric difference with this indicator in isolation. It is worth highlighting that the assessment of nutritional status includes a broad set of indicators, in addition to anthropometric measurements, including: food and nutrient intake; behavior; laboratory tests and physical examination^(27)^. In this study, although the infants presented adequate weight and height for age, a significant difference was identified in diet quality, which may represent a risk factor for negative health outcomes in children.

Corroborating, Ceni et al.^(10)^ when analyzing the eating practices and nutritional status of 45 children, aged six months to two years, and comparing them with the ten steps for healthy eating from the Food Guide for children under two years of age from the Ministry of Health^(3)^, observed that although most of the children evaluated were eutrophic, there was low adherence to exclusive breastfeeding and complementary feeding was inadequate, mainly due to the availability of ultra-processed foods. Therefore, the importance of public policies and greater actions to promote healthy eating in this age group is highlighted, in order to help families make better food choices, or even promote training for health professionals on this topic, with the aim of disseminating correct information that assists health services that serve this population.

The study showed a higher risk of PFD in the Non-Guided Group. One possible explanation for this finding could be the fact that the instrument used for assessment takes into account the perceptions of family members, who, because they have not received specialized guidance on infant feeding, may have a mistaken expectation regarding the quality and quantity of food that should be ingested by the child at this stage.

Eating disorders in children have a high prevalence, generating negative consequences for both the child and their family, since feeding is associated with the child's social, emotional, physical and cognitive development^(15)^. The EBAI proved to be a useful resource, as it allows for the screening of PFD, through responses from the child's main feeder, with quick application and which can be used by all health professionals. Early identification of children at risk of PFD allows professionals to make referrals more quickly, allowing for referral to a specialist, anticipating appropriate intervention and minimizing damage to development^(14)^.

The family environment proves to be fundamental in determining eating behavior, mothers with controlling attitudes and difficulty interacting with the child contribute to preferences, rejections and inadequate eating behaviors^(28)^. Santos et al.^(29)^, when evaluating the relationship between parental behavior during meals and the child's eating behavior, identified a significant association between parental practices with selective feeding of the child and food intake in the absence of hunger. The authors also reinforced that restrictive and pressured feeding practices used by parents caused children to increase their consumption of high-calorie foods, rich in fats and sugar, and decrease their intake of healthy foods, such as fruits and vegetables.

Despite the risk for PFD presented by infants in the Non-Oriented Group, the anthropometric parameters of most infants were adequate. These findings are in line with those evidenced by authors^(30)^ who did not identify changes in the anthropometric parameters of children with PFD, despite having the diagnosis of this condition. The aforementioned authors described that the main concerns of parents of children with PFD were: refusal to eat specific types or textures of food, difficulties in transitioning to age-appropriate foods, food selectivity, insufficient consumption of vegetables, fruits and legumes, refusal to eat, and inappropriate eating behaviors^(30)^. Thus, it is important to emphasize that the nutritional status of infants should not be observed in isolation by health professionals, without exploring the perceptions of parents and the conditions and quality of feeding.

It is considered that there is a need to reinforce the importance of specialized guidance at all levels of health care, in the first two years of a child's life, in order to help families make better food choices for children, promoting health and preventing complications.

Among the study's limitations are the self-reported data, which were provided by parents or caregivers, which can lead to reporting errors and memory bias. As well as the short follow-up period, since the reflection of the observed differences in food consumption and PFD may occur after 12 months of age. In addition, although both groups were within the age range established for the study, there was a statistical difference in their age at the time of data collection, with the Non-Guided Group having a higher average age at the time of data collection. However, this difference does not seem to compromise the results, as there are only a few days difference between them.

The content of the guidelines for introducing food provided by professionals working in childcare is unknown to the authors of this study, which constitutes a possible bias in the results. However, it was possible to verify that only one-third of the sample from the Non-Guided Group was instructed on complementary feeding, even though all were under regular medical follow-up. Thus, it is believed that the results presented may contribute to sensitizing colleagues to the importance of attentive conduct regarding the nutritional aspects of infants. Since the hospital's multidisciplinary outpatient clinic is an important pediatric referral center for families in the region, there was good participation in the study and no sample losses occurred.

## CONCLUSION

It was observed that nutritional and speech therapy guidance during the introduction of complementary feeding can play a relevant role in the quality of infants' diets, being associated with greater dietary diversity, higher consumption of foods rich in vitamin A, and lower intake of ultra-processed foods. Despite the differences observed in food consumption, there was a predominance of eutrophy in both groups. The risk of PFD was more frequent in the Non-Guided Group.
