# Supplementary material for: Nutritional and speech therapy guidance for the introduction of foods in infants: dietary profile and risk of pediatric feeding disorder
Source: Codas. 2026 Mar 27;38(2):e20240377. doi: 10.1590/2317-1782/e20240377en (PMC13075845; doi:10.1590/2317-1782/e20240377en)
Supplement: Translation

# Translation: Orientação nutricional e fonoaudiológica para introdução alimentar de lactentes: perfil alimentar e risco de distúrbio alimentar pediátrico

**DOI:** 10.1590/2317-1782/e20240377pt

**Authors:** Larissa Barz de Vargas, Giane Eichner, Bianca Nunes Pimentel, Giovana Cristina Ceni, Geovana de Paula Bolzan

**Affiliations:** 1 Programa de Pós-graduação em Distúrbios da Comunicação Humana, Universidade Federal de Santa Maria – UFSM - Santa Maria (RS), Brasil.; 2 Curso de Fonoaudiologia, Universidade Federal de Santa Maria – UFSM - Santa Maria (RS), Brasil.; 3 Departamento de Alimentos e Nutrição, Universidade Federal de Santa Maria – UFSM - Santa Maria (RS), Brasil.; 4 Departamento de Fonoaudiologia, Universidade Federal de Santa Maria – UFSM - Santa Maria (RS), Brasil.

**Keywords:** Comportamento Alimentar, Lactente, Nutrição do Lactente, Dieta, Avaliação Nutricional

## Abstract

**Objetivo:**

Avaliar o perfil alimentar, o estado nutricional e o risco de distúrbio alimentar pediátrico de lactentes saudáveis, em fase de introdução alimentar, e comparar estas variáveis entre grupos que receberam ou não orientação nutricional e fonoaudiológica especializada.

**Método:**

Estudo descritivo, observacional, transversal, com abordagem quantitativa. O Grupo Orientado foi composto por lactentes cujos pais relataram ter recebido orientação para a introdução alimentar em ambulatório multiprofissional. O Grupo Não Orientado foi composto por lactentes cujos pais não receberam orientação para introdução alimentar ou receberam na puericultura, por outros profissionais. Foram analisados prontuários e realizada entrevista e aferição de medidas antropométricas para traçar o perfil clínico e nutricional. A qualidade da dieta foi avaliada com o Formulário de Marcadores de Consumo Alimentar. A classificação do estado nutricional seguiu as curvas da World Health Organization. O risco de distúrbio alimentar pediátrico foi avaliado com a Escala Brasileira de Alimentação Infantil.

**Resultados:**

Participaram 60 lactentes. O Grupo Não Orientado iniciou a alimentação complementar precocemente, antes dos seis meses. A qualidade da alimentação do Grupo Orientado foi melhor, com maior diversidade alimentar, maior consumo de alimentos ricos em vitamina A e menor ingestão de alimentos ultraprocessados. Houve predomínio de eutrofia em ambos os grupos. O Grupo Orientado apresentou menor risco de distúrbio alimentar pediátrico.

**Conclusão:**

A orientação nutricional e fonoaudiológica para o início da alimentação complementar esteve associada à qualidade da dieta dos lactentes, porém não esteve associada ao estado nutricional. O risco de distúrbio alimentar pediátrico foi mais frequente no Grupo Não Orientado.

## INTRODUÇÃO

A nutrição tem papel extremamente relevante em todas as fases da vida, especialmente nos primeiros dois anos. Neste período é fundamental o incentivo e a adoção de hábitos alimentares saudáveis, que são decisivos para proporcionar o crescimento e desenvolvimento pleno^(1)^. O padrão alimentar estabelecido nesta faixa etária tende a permanecer e se consolidar na vida adulta. O consumo alimentar adequado, além de garantir as necessidades nutricionais, irá reduzir o risco de morbidade, principalmente desnutrição, excesso de peso, deficiência de micronutrientes e doenças associadas^(2)^.

A Organização Mundial da Saúde (OMS) recomenda o aleitamento materno exclusivo até os seis meses de vida e complementado até os dois anos ou mais, em virtude do leite materno ser inigualável e totalmente adaptado às necessidades nos primeiros meses de vida da criança^(2-4)^.

Porém, a partir dos seis meses, a alimentação deve ser complementada. A qualidade nutricional dos alimentos complementares é fundamental na prevenção da morbimortalidade na infância. A recomendação para iniciar a introdução alimentar com seis meses, leva em consideração a maturidade fisiológica e neuromuscular necessária para o lactente receber alimentos sólidos^(3,4,5,6)^. Nesta faixa etária, a criança já começa a estabelecer preferências alimentares. Além disso, é possível observar o desaparecimento do reflexo de protrusão de língua, melhor tolerância gastrointestinal e melhor capacidade de absorção de nutrientes^(2)^. A alimentação complementar precoce pode levar a sobrecarga renal, alergias alimentares, deficiências nutricionais, alteração na flora intestinal, infecção intestinal e respiratória e obesidade. Já a introdução alimentar tardia também poderá ter consequências como o retardo do crescimento e deficiências imunológicas^(2,7)^.

O Guia Alimentar para crianças brasileiras menores de dois anos, publicado pelo Ministério da Saúde (MS)^(3)^, reforça e divulga a importância da alimentação saudável, enfatizando os alimentos *in natura* ou minimamente processados como a base da alimentação da criança e de toda família, além de orientar a consistência adequada de acordo com a faixa etária. Apesar disso, autores evidenciaram o alto consumo de alimentos ultraprocessados entre crianças menores de um ano de idade, além da oferta de alimentos na consistência inadequada de acordo com a faixa etária, podendo causar repercussões negativas nas fases seguintes do desenvolvimento^(8)^.

O estado nutricional indica o resultado do equilíbrio entre o consumo de nutrientes e o gasto energético do organismo. A avaliação antropométrica é considerada um bom indicador de saúde, devido à facilidade de aplicação e padronização. Além disso, o baixo custo e amplitude dos aspectos analisados permite traçar o perfil nutricional individual ou de coletividades^(9)^. A avaliação do estado nutricional no primeiro ano de vida é fundamental, uma vez que neste período ocorre um crescimento acelerado^(10)^. Identificar precocemente lactentes em risco, com déficit ou excesso em seu crescimento, se torna imprescindível, já que fatores nutricionais influenciam diretamente o metabolismo, e podem acarretar consequências indesejáveis em longo prazo^(3,11)^.

O distúrbio alimentar pediátrico (DAP) é considerado uma condição complexa e multifatorial. Ele foi definido como ingestão oral prejudicada, que não é adequada para a idade, associada a no mínimo uma das seguintes disfunções: médica, nutricional, habilidade alimentar e/ou psicossocial^(12)^. Esse distúrbio pode ocorrer em qualquer fase da infância, sendo mais prevalente ao redor dos seis meses até os quatro anos de vida^(12-13)^. Estudos apontam a prevalência de DAP de 20% a 35% entre crianças com desenvolvimento intelectual normal^(14-15)^.

Em razão da complexidade, é indispensável uma equipe interdisciplinar para o diagnóstico, tratamento e acompanhamento do DAP^(14)^. Vale ressaltar que o ato de comer transcende questões anatômicas e fisiológicas, pois está inserido em um contexto social e cultural, desta maneira está associado com o crescimento e desenvolvimento social, emocional e cognitivo. A identificação precoce deste distúrbio por profissionais da saúde pode minimizar e solucionar problemas alimentares, através de um tratamento precoce e especializado^(15)^.

O período de introdução da alimentação complementar leva a repercussões em todas as fases da vida, porém poucos estudos foram realizados quanto à alimentação e nutrição dos lactentes durante a introdução alimentar. Assim, o objetivo do presente estudo foi avaliar o perfil alimentar, o estado nutricional e o risco de DAP de lactentes saudáveis, em fase de introdução alimentar, bem como comparar estas variáveis entre grupos que receberam ou não orientação nutricional e fonoaudiológica especializada.

## MÉTODO

Trata-se de um estudo descritivo, observacional, transversal, com abordagem quantitativa. O projeto de pesquisa foi previamente aprovado no comitê de ética da instituição de origem sob parecer de número 5.861.862.

O estudo foi realizado com lactentes a termo, acompanhados em ambulatório pediátrico multiprofissional no Hospital Universitário de Santa Maria, hospital de nível terciário, que serve como referência para a região central do Rio Grande do Sul. Foram incluídos na amostra lactentes de sete a 12 meses de idade, que já tivessem iniciado a alimentação complementar há no mínimo um mês e que estivessem em alimentação exclusivamente por via oral. Foram excluídos lactentes com diagnóstico de doenças neurológicas, cardiológicas, malformações craniofaciais ou de vias aerodigestivas, ou com qualquer outra condição clínica que interferisse diretamente na segurança da deglutição e na eficácia da alimentação oral. Todos os participantes tiveram o Termo de Consentimento Livre e Esclarecido assinado pelos responsáveis. A coleta de dados foi realizada de outubro de 2022 a setembro de 2023.

O cálculo amostral foi realizado a partir de estudo piloto com dez sujeitos atendidos no ambulatório de alimentação infantil. O parâmetro utilizado para este cálculo foi o escore T da Escala Brasileira de Alimentação Infantil. Considerou-se um nível de significância (α) de 95% e um erro de amostragem de 5% e chegando-se a um tamanho amostral mínimo de 60 lactentes, permitindo comparações entre os subgrupos formados conforme a exposição à orientação especializada. A categorização posterior dos participantes em dois subgrupos, orientado e não orientado, foi realizada com base nas informações obtidas durante a coleta de dados, e serviu para fins analíticos e comparativos. O Grupo Orientado foi composto por lactentes cujos os pais receberam orientações nutricionais e fonoaudiológicas especializadas para introdução alimentar. O Grupo Não Orientado foi composto por lactentes cujos pais iniciaram a introdução alimentar por conta própria ou conforme orientação na puericultura, por médico ou enfermeiro. As comparações entre os grupos buscaram identificar associações entre a orientação especializada e as variáveis estudadas, como qualidade da alimentação, estado nutricional e risco para DAP, de forma compatível, que não permite inferência de causalidade. As exposições e desfechos foram coletados simultaneamente e as comparações foram feitas com base nas condições observadas no momento da coleta.

A amostra foi recrutada no Ambulatório multiprofissional pediátrico do hospital. Os responsáveis foram convidados a participar do estudo por meio de contato telefônico e foram agendados atendimentos presenciais no hospital. Foram convidadas as famílias que haviam feito atendimento especializado para introdução alimentar no ambulatório para participarem do Grupo Orientado. Para o Grupo Não Orientado foram convidadas as famílias atendidas no ambulatório por outros motivos, tais como avaliação de frênulo lingual; avaliação auditiva; manejo clínico do aleitamento materno ao nascimento e participação no grupo de intervenção musical para lactentes, e que não haviam realizado atendimento fonoaudiológico e/ou nutricional para a dieta complementar.

O atendimento para orientações quanto à introdução alimentar, nas quais participaram os lactentes do Grupo Orientado, são rotineiramente praticados no ambulatório extensionista multiprofissional. Este atendimento especializado é oferecido a todas as famílias que comparecem para atendimento logo após o nascimento dos bebês, sendo facultado a elas a decisão de agendar o atendimento.

As orientações são realizadas aos pais dos lactentes, em conjunto entre nutricionista e fonoaudióloga e/ou estudante de Fonoaudiologia sob supervisão acadêmica. As orientações nutricionais e fonoaudiológicas que são praticadas nestes atendimentos são baseadas no livro Relações Cognitivas com o alimento na infância^(16)^ e no Guia Alimentar para crianças brasileiras menores de dois anos^(3)^. São abordados os seguintes tópicos: aleitamento materno continuado; grupos alimentares, fracionamento das refeições; quantidade de alimento a ser ofertado; sinais de fome e saciedade; mitos e crenças da alimentação complementar; consequências da introdução alimentar precoce ou tardia; métodos de oferta de alimentos (tradicional, BLW (*Baby-Led Weaning*) e misto); consistência adequada dos alimentos; utensílios adequados; posicionamento da criança; além da interação de familiares no momento da oferta de alimentos. As orientações são explicadas oralmente e são entregues por escrito, em material que contém texto e imagens. Além disso, os participantes recebem o contato de um canal permanente para tirarem dúvidas com os profissionais, se necessitarem, via rede social.

A coleta de dados envolveu a investigação de dados clínicos e sociodemográficos, aplicação de protocolos de rastreio para perfil de consumo alimentar, avaliação nutricional e para risco de DAP. Primeiramente, foram coletadas informações dos prontuários referentes às seguintes variáveis: idade (em meses), sexo (feminino ou masculino), cor (branca e não branca), idade da mãe (em anos), peso (kg) e estatura (cm) do nascimento. Posteriormente, foi realizada uma entrevista para coletar dados sobre a renda familiar, rede de apoio, aleitamento materno, idade de início da introdução alimentar, se o responsável recebeu orientação de algum profissional referente à introdução alimentar, tipo de alimento ofertado inicialmente e se a criança apresentou alguma dificuldade para introdução alimentar.

A qualidade da alimentação dos lactentes foi avaliada utilizando-se o Formulário de Marcadores de Consumo Alimentar para crianças menores de dois anos de idade do MS. Esse instrumento tem a finalidade de conhecer o padrão do consumo alimentar por meio de questões aplicadas aos pais conforme a faixa etária do lactente. As questões permitem caracterizar a introdução da alimentação complementar, identificar o tipo de alimentação atual e verificar a adoção de comportamento alimentar de risco para o desenvolvimento de anemia e obesidade infantil^(17)^.

As variáveis de consumo alimentar foram organizadas com base nos principais grupos de alimentos e comportamentos de risco identificados no formulário. Os grupos de alimentos incluem frutas, legumes, carnes, grãos, e laticínios, sendo categorizados quanto à presença ou ausência de consumo no dia anterior ao inquérito. Além disso, é considerada a frequência de ingestão diária e a forma de oferta dos alimentos (exemplo: em pedaços, amassada, passada em peneira, liquidificada ou só o caldo). Em relação aos comportamentos de risco, foram identificadas variáveis como consumo de açúcares, sucos adoçados e alimentos ultraprocessados, além da ingestão de alimentos ricos em ferro, categorizadas como presença ou ausência. Essas variáveis permitem uma caracterização abrangente da qualidade da alimentação e dos alimentos.

A avaliação do estado nutricional foi realizada por nutricionista experiente. Foram aferidos o peso, em balança pediátrica digital (Balmak^®^) com graduação de 0.010 kg e capacidade para 15 kg, e o comprimento, com estadiômetro infantil (Avanutri^®^) com graduação de 1mm. Para classificação do estado nutricional dos lactentes foram utilizadas as curvas da *World Health Organization* de 2006^(18)^, por meio do software *Who Anthro*, de acordo com o escore Z.

Para avaliar o risco de DAP, foi aplicada a Escala Brasileira de Alimentação Infantil (EBAI). Este instrumento de rastreio é destinado a crianças na faixa etária de 6 meses a 5 anos e 11 meses e foi recentemente validado para a população brasileira. A EBAI é composta por quatorze questões relacionadas à alimentação da criança, direcionada aos responsáveis. As perguntas compreendem os domínios de alimentação: motor oral, sensorial oral, apetite e preocupações dos pais com a alimentação. A presença ou ausência de risco de DAP é calculada por meio de um escore ao final do questionário. Quando o escore total for de até 60 é considerada ausência de risco de DAP; escores de 61 a 65 indicam risco leve; escores de 66 a 70, indicam risco moderado; e escores acima de 70 indicam risco severo^(14)^.

Todos os instrumentos de rastreio foram aplicados por uma nutricionista e por uma fonoaudióloga, previamente treinadas. Os dados foram inseridos em uma planilha no programa Excel®, versão 2010 para Windows, e, posteriormente, exportados para o programa Statistica versão 9.1. Foi realizada a análise das variáveis estudadas, com apresentação de médias e desvios-padrão ou proporções, conforme a natureza da variável. As análises de associação foram realizadas pelos testes de qui-quadrado de Pearson ou Exato de Fisher. Para verificar as diferenças de médias foi utilizado o teste t-Student. O nível de significância para todas as análises foi de 5% (p<0.05).

## RESULTADOS

Participaram do estudo 60 lactentes a termo. A média de idade da amostra foi de 8.95±1.75 meses. A maioria era do sexo masculino (63.3%), cor branca (80%) e vivia em família com rede de apoio (85%). A média de idade gestacional ao nascer foi de 38.1 ± 1.21 semanas, e a idade materna média foi de 30.3 ± 6.7 anos. A renda per capita média da amostra foi de R$ 627.73 ± 252,60.

Quando estratificados por relato de recebimento de orientação nutricional e fonoaudiológica especializada para a introdução alimentar, observou-se que o Grupo Orientado apresentou média de idade de 9.4 ± 2.04 meses, enquanto o Grupo Não Orientado tinha média de 8.5 ± 1.30 meses (p = 0.018). Embora tenha sido identificada uma diferença estatística na idade entre os subgrupos, optou-se por não realizar análise multivariada neste estudo em razão do tamanho da amostra. No entanto, a proximidade entre as médias (9.4 vs. 8.5 meses) sugere que essa diferença etária não compromete a interpretação dos resultados observados.

A Tabela 1 apresenta detalhadamente as características demográficas, socioeconômicas e de apoio familiar da amostra total e conforme relato de recebimento de orientação especializada.

**Tabela 1:** Características demográficas, socioeconômicas e de apoio familiar da amostra total e conforme relato de recebimento de orientação especializada (N = 60)

**Variáveis**	Grupos				
	**Orientado (N=30)**		**Não Orientado (N=30)**		
	**N (%)**	**Média ± DP**	**N (%)**	**Média ± DP**	**p**
**Idade gestacional ao nascer (semanas)**		37.88 ± 1.14		38.33 ± 1.26	0.605*
**Idade atual (meses)**		9.40 ± 2.04		8.50 ± 1.30	0.018*
**Idade materna**		30.13 ± 6.88		30.46 ± 6.68	0.875*
**Renda per capita (reais)**		638.00 ± 241.41		617.46 ± 264.94	0.619*
**Sexo**					
Feminino	9 (30.00)		13 (43.33)		0.283**
Masculino	21 (70.00)		17 (56.67)		
**Cor**					
Branca	25 (83.33)		23 (76.67)		0.518**
Não branca	5 (16.67)		7 (23.33)		
**Rede de apoio**					
Com	28 (93.33)		23 (76.67)		0.072**
Sem	2 (6.67)		7 (23.33)		

* Teste t-Student;

** Teste Qui quadrado de Pearson

Legenda: DP = Desvio Padrão; N = número de participantes; % = porcentagem de participantes

Em relação à alimentação complementar, a média de idade de início foi de 6.16±0.46 meses no Grupo Orientado e de 5.46±1.10 meses no Grupo Não Orientado. Na Tabela 2 estão as características alimentares dos lactentes na fase de introdução alimentar. Foi verificado predomínio do aleitamento misto no Grupo Orientado e do aleitamento artificial no Grupo Não Orientado, ao iniciarem a dieta complementar. As frutas e comida de sal foram os alimentos escolhidos para serem ofertados inicialmente, não sendo constatadas dificuldades para a introdução da dieta complementar em ambos os grupos. Verificou-se que 63.33% das famílias dos lactentes do Grupo Não Orientado não receberam orientações específicas quanto à introdução da alimentação complementar.

**Tabela 2:** Características alimentares dos lactentes durante a fase de introdução alimentar conforme relato de recebimento de orientação (N = 60)

Variáveis	Grupos		
	Orientado (N=30)	Não Orientado (N=30)	
	N (%)	N (%)	p
**Tipo de alimento ofertado inicialmente**			
Fruta	10 (33.33)	7 (23.33)	
Comida de sal	11 (36.67)	11 (36.67)	0.843**
Frutas e comida de sal	11 (36.67)	12 (40.00)	
**Profissional que orientou IA**			
Nutricionista e fonoaudióloga	30 (100.00)	0 (0.00)	0.001**
Outro profissional na puericultura	0 (0.00)	11 (36.66)	
Não recebeu orientação	0 (0.00)	19 (63.33)	
**Dificuldades para IA**			
Sim	12 (40.00)	13 (43.33)	0.793**
Não	18 (60.00)	17 (56.67)	
**Tipo de aleitamento materno até a IA**			
AA	9 (30.00)	19 (63.33)	
AMM	20 (66.67)	9 (30.00)	0.017**
AME	1 (3.33)	2 (6.67)	

** Teste Qui quadrado de Pearson

Legenda: N = número de participantes; % = porcentagem de participantes; IA = introdução alimentar; AA = aleitamento artificial; AMM = aleitamento materno misto; AME = aleitamento materno exclusivo

A qualidade da alimentação complementar do Grupo Orientado foi significativamente melhor comparada à do Grupo Não Orientado. O Grupo Orientado apresentou maior diversidade alimentar e maior consumo de alimentos ricos em vitamina A (legumes, vegetais ou frutas de cor alaranjada), e o Grupo Não Orientado apresentou alto consumo de alimentos ultraprocessados (Tabela 3).

**Tabela 3:** Qualidade da alimentação na fase de introdução alimentar dos lactentes, avaliada pelos marcadores de consumo alimentar, conforme relato de recebimento de orientação (N = 60)

**Variáveis**	**Grupos**		
	**Orientado** **(N=30)**	**Não Orientado (N=30)**	
	**N (%)**	**N (%)**	**p**
**Alimentação geral**			
Adequada	24 (80.00)	21 (70.00)	0.001**
Inadequada	6 (20.00)	9 (30.00)	
**Aleitamento materno continuado**			
Sim	18 (60.00)	13 (43.33)	0.196**
Não	12 (40.00)	17 (56.67)	
**Introdução de alimentos**			
Sim	19 (63.33)	16 (53.33)	0.432**
Não	11 (36.67)	14 (46.67)	
**Diversidade alimentar mínima**			
Sim	22 (73.33)	13 (43.33)	0.018**
Não	8 (26.67)	17 (56.67)	
**Frequência alimentar mínima**			
Sim	22 (73.33)	21 (70.00)	0.774**
Não	8 (26.67)	9 (30.00)	
**Consumo de alimentos ricos em ferro**			
Sim	27 (90.00)	23 (76.67)	0.149***
Não	3 (10.00)	7 (23.33)	
**Consumo de alimentos ricos em vitamina A**			
Sim	28 (93.33)	20 (66.67)	0.010***
Não	2 (6.67)	10 (33.33)	
**Consumo de alimentos ultraprocessados**			
Sim	6 (20.00)	23 (76.67)	0.001**
Não	24 (80.00)	7 (23.33)	
**Hambúrgueres/embutidos**			
Sim	0 (0.00)	22 (73.33)	0.001***
Não	30 (100.00)	8 (26.67)	
**Bebidas adoçadas**			
Sim	2 (6.67)	10 (33.33)	0.010***
Não	28 (93.33)	20 (66.67)	
**Macarrão instantâneo, salgadinhos de pacote e biscoitos salgados**			
Sim	1 (3.33)	11 (36.67)	0.001***
Não	29 (96.67)	19 (63.33)	
**Biscoito recheado, doces e guloseimas**			
Sim	4 (13.33)	18 (60.00)	0.001***
Não	26 (86.67)	12 (40.00)	

** Teste Qui quadrado de Pearson;

*** Teste Exato de Fisher

Legenda: N = número de participantes; % = porcentagem de participantes

Na Figura 1 pode ser observada diferença significativa entre o consumo de alimentos, em que o Grupo Orientado apresentou maior consumo de alimentos *in natura* ou minimamente processados (arroz, batata, inhame, feijão, fígado, carnes, verduras e legumes) e o Grupo Não Orientado apresentou maior ingestão de alimentos ultraprocessados (doces e biscoitos recheados; macarrão, salgadinhos e biscoitos; bebidas adoçadas e hambúrgueres/embutidos).

**Figura 1:** Padrão alimentar da amostra, conforme os grupos

Em relação ao estado nutricional (Tabela 4), apresentando em escore Z, foi observada a prevalência de eutrofia no nascimento e no momento da coleta de dados, de acordo com o indicador peso para estatura, em ambos os grupos.

**Tabela 4:** Características antropométricas e estado nutricional de lactentes no nascimento e na fase de introdução alimentar, conforme relato de recebimento de orientação (N = 60)

**Variáveis**	**Grupos**				
	**Orientado (N=30)**		**Não Orientado (N=30)**		
	**N (%)**	**Média ± DP**	**N (%)**	**Média ± DP**	**p**
Peso ao nascer (kg)		2.96 ± 0.54		3.06 ± 0.44	0.253*
Estatura ao nascer (cm)		46.92 ± 2.65		47.97 ± 2.32	0.489*
Peso atual (kg)		8.85 ± 1.32		8.96 ± 1,29	0.909*
Estatura atual (cm)		71.50 ± 4.53		73.48 ± 5,66	0.242*
**Peso para estatura nascimento**					
Magreza acentuada	8 (26.67)		4 (13.33)		0.353**
Magreza	1 (3.33)		0 (0.00)		
Eutrofia	13 (43.33)		21 (70.00)		
Risco de sobrepeso	5 (16.67)		5 (16.67)		
Sobrepeso	2 (6.67)		0 (0.00)		
Obesidade	1 (3.33)		0 (0.00)		
**Peso para estatura atual**					
Magreza acentuada	0 (0.00)		1 (3.33)		0.891**
Magreza	0 (0.00)		3 (10.00)		
Eutrofia	26 (86.21)		23 (76.67)		
Risco de sobrepeso	1 (3.45)		3 (10.00)		
Sobrepeso	3 (10.34)		0 (0.00)		
Obesidade	0 (0.00)		0 (0.00)		
**Peso na idade nascimento**	5 (16.66)		0 (0.00)		
Muito baixo	1 (3.33)		5 (16.66)		0.600**
Baixo	24 (80.00)		25 (83.33)		
Adequado	0 (0.00)		0 (0.00)		
Elevado					
**Peso na idade atual**					
Muito baixo	0 (0.00)		0 (0.00)		
Baixo	1 (3.33)		1 (3.33)		0.561**
Adequado	25 (83.33)		28 (93.33)		
Elevado	3 (10.00)		1 (3.33)		
**Estatura para idade atual**					
Muito baixa	0 (0.00)		0 (0.00)		
Baixa	3 (10.00)		0 (0.00)		0.705**
Adequada	27 (90.00)		30 (0.00)		

* Teste t-Student;

** Teste Qui quadrado de Pearson

Legenda: DP = Desvio Padrão; N = número de participantes; % = porcentagem de participantes

Foi identificada maior frequência de risco de DAP no Grupo Não Orientado sendo classificado com risco leve a moderado (Tabela 5).

**Tabela 5:** Associação do risco de distúrbio alimentar pediátrico nos lactentes com os grupos e frequência da classificação do risco nos grupos estudo e controle (N = 60)

**Variáveis**	**Grupos**		
	**Orientado (N=30)**	**Não Orientado (N=30)**	
	**N (%)**	**N (%)**	**p**
**Risco de DAP**			
Sim	1 (3.33)	10 (33.33)	0.001**
Não	29 (96.67)	20 (66.67)	
**Classificação do risco de DAP**			
Ausência de risco	29 (96.67)	20 (66.67)	-
Leve	1 (3.33)	7 (23.33)	
Moderado	0 (0.00)	3 (10.00)	
Grave	0 (0.00)	0 (0.00)	

** Teste Qui-quadrado de Pearson

Legenda: N = número de participantes; % = porcentagem de participantes; DAP = distúrbio alimentar pediátrico

## DISCUSSÃO

De acordo com o MS, a introdução alimentar deve ser iniciada aos seis meses de idade, de forma lenta e gradual. Neste período, as necessidades por energia e nutrientes do lactente excedem o que pode ser provido pelo aleitamento materno exclusivo, além do lactente começar a demonstrar os sinais de prontidão, que propiciam o início da oferta de alimentos. A alimentação complementar apresenta uma relação extremamente relevante no desenvolvimento físico, neurológico e motor da criança e repercute na formação de hábitos alimentares saudáveis, que persistem até a vida adulta^(3)^.

Neste estudo, observou-se maior frequência de aleitamento artificial e início precoce da alimentação complementar no Grupo Não Orientado. Este achado corrobora o que foi evidenciado em estudo retrospectivo, cujos autores, ao caracterizarem a alimentação complementar de mais de 700 lactentes, verificaram que a média de idade para introdução de alimentos foi de 5,5 meses^(19)^. Carneiro et al.^(20)^ também observaram, em seu estudo, que 66,66% dos lactentes iniciaram a alimentação complementar precocemente, entre quatro e cinco meses de idade, e os motivos informados pelos pais para esse início foram: o uso de fórmula infantil, retorno ao trabalho, baixo ganho de peso e opção materna. Autores^(6)^ afirmam que os pais de lactentes optam por oferecer outros alimentos, sentindo-se mais seguros por terem maior controle do que está sendo ingerido pela criança. A explicação para isso seria a falta de orientação ou de compreensão sobre os processos fisiológicos e hormonais da produção do leite materno e também por não conseguirem ter o controle sobre a quantidade de leite materno ingerido^(6)^.

No entanto, a introdução precoce de alimentos complementares pode ocasionar uma série de prejuízos. Inicialmente, essa prática leva à diminuição do aleitamento materno, podendo levar à interrupção do desenvolvimento motor-oral equilibrado, que ocorre durante a amamentação, com possíveis consequências para as funções de respiração, mastigação, deglutição e fala^(2)^. Do ponto de vista nutricional, a introdução alimentar precoce da alimentação complementar pode interferir na absorção de nutrientes importantes obtidos do leite materno, podendo ainda ocasionar aumento no risco de contaminação, reações alérgicas e doenças atópicas^(11)^. Ainda, a experiência nutricional precoce pode acarretar um efeito duradouro, persistente ao longo da vida, predispondo o sujeito a determinadas doenças^(11)^.

Neste estudo, a orientação nutricional e fonoaudiológica esteve associada a melhores indicadores na introdução da alimentação complementar. Os lactentes cujos pais foram orientados em atendimento específico para esse fim, Grupo Orientado, iniciaram a introdução alimentar com a idade recomendada, apresentando melhor qualidade da dieta e menor risco de DAP. O nutricionista e o fonoaudiólogo, assim como os demais profissionais de saúde desempenham papel importante no aconselhamento das famílias, uma vez que o comportamento alimentar da criança é consequência das atitudes dos pais^(21)^. A educação em saúde em tempo oportuno, reforçando a superioridade do leite materno e ressaltando a importância de uma introdução alimentar adequada, com a utilização de estratégias lúdicas e dinâmicas, propicia às famílias maior autonomia e formação de hábitos alimentares saudáveis nas crianças^(22)^.

Em relação à qualidade da dieta dos lactentes, de acordo com os marcadores do consumo alimentar, proposto pelo Ministério da Saúde *^(^* ^17)^, o Grupo Orientado apresentou maior frequência de diversidade alimentar (mais de quatro grupos de alimentos) e maior consumo de alimentos ricos em vitamina A, caracterizando um padrão alimentar mais adequado. Resultados semelhantes foram observados por outros autores que desenvolveram um ensaio controlado randomizado com lactentes de seis a 12 meses e suas mães^(23)^. Eles observaram que o grupo que obteve educação nutricional com foco na alimentação complementar apresentou maior diversidade alimentar^(23)^. Tais achados suportam a importância da orientação nutricional para lactentes, uma vez que eles podem apresentar uma série de deficiências nutricionais, principalmente anemia ferropriva. E, além disso, reforçam a necessidade de fornecer micronutrientes advindos da alimentação complementar^(2)^.

Sugere-se que a relação positiva das orientações realizadas ao Grupo Orientado pode ter ocorrido devido ao acolhimento e orientação aos pais dos lactentes sobre como proceder durante o momento de início da alimentação complementar. Destaca-se, ainda, que o ambulatório onde as orientações são realizadas conta com um canal digital, onde as famílias podem solucionar suas dúvidas, o que pode ter colaborado com a segurança dos familiares no momento da introdução alimentar. De acordo com Vieira et al.^(24)^, o diálogo proporciona a construção de vínculos com os educandos, facilitando a interação no movimento ensino-aprendizagem, contribuindo para a disseminação de conhecimento sobre a alimentação saudável e resultando em uma boa adesão às orientações.

Em contrapartida, no Grupo Não Orientado constatou-se a introdução de alimentos ultraprocessados precocemente, dentre eles: hamburguer/embutidos, bebidas adoçadas, macarrão instantâneo, salgadinhos de pacote, biscoitos salgados, biscoitos recheados, doces e guloseimas, apresentando diferença significativa entre os grupos, o que pode estar associado ao padrão dietético contemporâneo. Cabe destacar que o Guia Alimentar para menores de dois anos do Ministério da Saúde^(3)^, recomenda a adição mínima de sal nas preparações ofertadas e a introdução de alimentos ricos em açúcar ou preparações com adição de açúcar apenas a partir de dois anos de idade^(3)^. A ingestão de alimentos com altas taxas de calorias, sódio, açúcares e gorduras, geram consequência a curto e longo prazo, estando associado ao excesso de peso, cáries dentárias, deficiência de micronutrientes, dislipidemias, hipertensão arterial e diabetes^(1)^.

Na mesma direção, outros estudos evidenciaram a prevalência de introdução precoce de alimentos de alta densidade calórica e de baixa qualidade nutricional^(25)^, e apontaram que mais da metade das crianças antes de um ano de vida ingeriram doces, achocolatados e macarrão instantâneo *^(^* ^11)^, além do alto consumo de frituras, refrigerantes e sal^(1)^. Dados do Sistema de Vigilância Alimentar e Nutricional (SISVAN) em 2022, também demonstraram o consumo precoce de alimentos ultraprocessados de crianças entre seis e 23 meses de idade, sendo que 44% consumiram alimentos ultraprocessados, 12% alimentos embutidos, 22% macarrão instantâneo, salgadinhos ou biscoitos salgados, 25% biscoitos recheados, doces ou guloseimas e 28% bebidas adoçadas *^(^* ^26)^.

Em relação ao estado nutricional, foi observado que ambos os grupos, no nascimento e na coleta de dados, apresentaram prevalência de eutrofia, não havendo diferença antropométrica com este indicador de forma isolada. Vale destacar, que a avaliação do estado nutricional inclui um conjunto amplo de indicadores, além das medidas antropométricas, dentre eles: a ingestão de alimentos e nutrientes; comportamento; exames laboratoriais e exame físico^(27)^. Neste estudo, apesar dos lactentes terem apresentado peso e estatura adequados para idade, foi identificada uma diferença significativa na qualidade da dieta, a qual pode representar um fator de risco para desfechos negativos na saúde das crianças.

Corroborando, Ceni et al. ^(10)^ ao analisarem as práticas alimentares e o estado nutricional de 45 crianças, de seis meses a dois anos de idade, e compararem com os dez passos para uma alimentação saudável do Guia Alimentar para menores de dois anos do Ministério da Saúde^(3)^, observaram que apesar da maioria das crianças avaliadas serem eutróficas, houve baixa adesão ao aleitamento materno exclusivo (AME) e a alimentação complementar estava inadequada, principalmente devido à oferta de alimentos ultraprocessados. Logo, destaca-se a importância de políticas públicas e maiores ações para promover a promoção da alimentação saudável nesta faixa etária, com o intuito de auxiliar os familiares a fazerem melhores escolhas alimentares, ou ainda promover capacitações para profissionais da saúde sobre esta temática, com o objetivo de difundir informações corretas, que auxiliem os serviços de saúde que atendem este público.

Observou-se no estudo o maior risco de DAP no Grupo Não Orientado. Uma possível explicação para esse achado poderia ser o fato de o instrumento utilizado para avaliação levar em consideração as percepções dos familiares, que por não terem recebido orientações especializadas sobre a alimentação dos lactentes, podem ter uma expectativa equivocada quanto à qualidade e quantidade de alimento que deveria ser ingerido pela criança nesta fase.

Os distúrbios alimentares em crianças apresentam alta prevalência, gerando consequências negativas tanto para a criança quanto para sua família, uma vez que a alimentação está associada ao desenvolvimento social, emocional, físico e cognitivo da criança^(15)^. A EBAI mostrou-se ser um recurso útil, em virtude de possibilitar o rastreamento do DAP, por meio de respostas pelo principal alimentador da criança, com rápida aplicação e que pode ser utilizada por todos os profissionais de saúde. A identificação precoce de crianças em risco de DAP permite aos profissionais realizarem encaminhamentos com mais agilidade, permitindo o direcionamento para um especialista, antecipando a adequada intervenção e minimizando danos ao desenvolvimento^(14)^.

O âmbito familiar mostra-se fundamental na determinação do comportamento alimentar, mães com posturas controladoras e com dificuldade de interagir com a criança contribuem para preferências, rejeições e comportamentos alimentares inadequados^(28)^. Santos et al.^(29)^, ao avaliar a relação entre o comportamento dos pais durante a refeição e o comportamento alimentar da criança, identificaram associação significativa entre as práticas parentais com alimentação seletiva do filho e a ingestão de alimentos na ausência de fome. Os autores reforçaram ainda que práticas de restrição e pressão alimentar utilizadas pelos pais provocaram nas crianças aumento do consumo de alimentos calóricos, ricos em gorduras e açúcar, e diminuição na ingestão de alimentos saudáveis, como frutas e verduras.

Apesar do risco para DAP apresentado pelos lactentes do Grupo Não Orientado, os parâmetros antropométricos da maioria dos lactentes estiveram adequados. Tais achados vão ao encontro do evidenciado por autores^(30)^ que não identificaram alterações nos parâmetros antropométricos de crianças com DAP, apesar de terem o diagnóstico dessa condição. Os referidos autores descreveram que as principais preocupações dos pais de crianças com DAP foram: recusa em ingerir tipos ou texturas específicas de alimentos, dificuldades na transição para alimentos adequados à idade, seletividade alimentar, consumo insuficiente de hortaliças, frutas e leguminosas, recusa em se alimentar, e comportamentos alimentares inadequados^(30)^. Assim, cabe ressaltar que o estado nutricional dos lactentes não deve ser observado de forma isolada pelos profissionais da saúde, sem que sejam exploradas as percepções dos pais e as condições e a qualidade da alimentação.

Considera-se que há necessidade de reforçar a importância de orientações especializadas em todos os níveis de atenção à saúde, nos dois primeiros anos de vida da criança, com o intuito de auxiliar as famílias a fazerem melhores escolhas alimentares para as crianças, promovendo saúde e prevenindo agravos.

Dentre as limitações do estudo estão os dados autorrelatados, que foram fornecidos pelos pais ou cuidadores, o que pode levar a erros de relato e viés de memória. Bem como, o curto período de acompanhamento, já que o reflexo das diferenças observadas no consumo alimentar e o DAP podem ocorrer após 12 meses de idade. Além disso, apesar de ambos os grupos estarem na faixa etária estabelecida para o estudo, houve diferença estatística na idade deles no momento da coleta de dados, com o Grupo Não Orientado, que apresentava maior média de idade no momento da coleta de dados. Contudo, esta diferença parece não comprometer os resultados, por serem poucos dias de diferença entre eles.

O conteúdo das orientações para introdução alimentar realizadas pelos profissionais que atuam na puericultura é desconhecido pelos autores do presente estudo, o que constitui um possível viés nos resultados. Porém foi possível verificar que apenas um terço da amostra do Grupo Não Orientado foi instruída para a dieta complementar, mesmo que todos estivessem em acompanhamento médico regular. Assim acredita-se que os resultados apresentados poderão contribuir para sensibilizar os colegas para a importância de uma conduta atenta aos aspectos alimentares de lactentes.

Como o ambulatório multiprofissional do hospital é uma referência importante em pediatria para as famílias da região, houve boa adesão ao estudo e não ocorreram perdas amostrais.

## CONCLUSÃO

Observou-se que a orientação nutricional e fonoaudiológica na introdução da alimentação complementar pode ter papel relevante na qualidade da dieta dos lactentes, esteve associada à maior diversidade alimentar, maior consumo de alimentos ricos em vitamina A e menor ingestão de alimentos ultraprocessados. Apesar das diferenças observadas no consumo alimentar, houve predomínio de eutrofia em ambos os grupos. O risco de DAP foi mais frequente no Grupo Não Orientado.
